# Comparative Accuracy of ChatGPT 4.0 and Google Gemini in Answering Pediatric Radiology Text-Based Questions

**DOI:** 10.7759/cureus.70897

**Published:** 2024-10-05

**Authors:** Mohammed Abdul Sami, Mohammed Abdul Samad, Keyur Parekh, Pokhraj P Suthar

**Affiliations:** 1 Department of Diagnostic Radiology and Nuclear Medicine, Rush University Medical Center, Chicago, USA; 2 Department of Diagnostic Radiology, Des Moines University College of Osteopathic Medicine, West Des Moines, USA

**Keywords:** ai in medical education, chatgpt, google gemini, large language models (llms), pediatric radiology

## Abstract

Aims and objectives: This study evaluates the accuracy of two AI language models, ChatGPT 4.0 and Google Gemini (as of August 2024), in answering a set of 79 text-based pediatric radiology questions from “Pediatric Imaging: A Core Review.” Accurate interpretation of text and images is critical in radiology, making AI tools valuable in medical education.

Methods: The study involved 79 questions selected from a pediatric radiology question set, focusing solely on text-based questions. ChatGPT 4.0 and Google Gemini answered these questions, and their responses were evaluated using a binary scoring system. Statistical analyses, including chi-square tests and relative risk (RR) calculations, were performed to compare the overall and subsection accuracy of the models.

Results: ChatGPT 4.0 demonstrated superior accuracy, correctly answering 83.5% (66/79) of the questions, compared to Google Gemini's 68.4% (54/79), with a statistically significant difference (p=0.0255, RR=1.221). No statistically significant differences were found between the models within individual subsections, with p-values ranging from 0.136 to 1.

Conclusion: ChatGPT 4.0 outperformed Google Gemini in overall accuracy for text-based pediatric radiology questions, highlighting its potential utility in medical education. However, the lack of significant differences within subsections and the exclusion of image-based questions underscore the need for further research with larger sample sizes and multimodal inputs to fully assess AI models' capabilities in radiology.

## Introduction

The rapid innovation of artificial intelligence in the 21st century has brought about language learning models, also known as large language models (LLMs), as a tool with multiple capabilities [1-3]. These LLMs build on deep learning algorithms that allow them to analyze inputs and create text by predicting sequences of words [1, 4, 5]. The models are trained on substantial sets of data, enabling them to have an extensive understanding of language and perform various tasks based on their inputs, such as answering questions [2, 3]. Particularly, two leading language learning models are OpenAI's ChatGPT and Google's Gemini. ChatGPT, based on its recent GPT-4 architecture, excels in tasks that require logical text creation [6, 7]. Its development is part of OpenAI's refinements of its model, building on earlier versions such as ChatGPT 3.0 and ChatGPT 3.5 [2, 8]. Similarly, Google Gemini represents Google's AI model that is designed to complete multimodal tasks by integrating inputs from text, images, and video responses [1, 4, 9]. Although both models have their similarities, ChatGPT and Gemini have distinct differences. ChatGPT is often utilized to handle inputs based on reasoning and discussion (although it can still analyze images), whereas Google Gemini relies on its ability to both input and output a broader range of responses [1, 10, 11].

This study aims to compare ChatGPT and Google Gemini in their abilities to accurately answer questions from a pediatric radiology-specific resource. By evaluating their performance on a standardized set of questions, we aim to determine if there is a significant difference in accuracy between ChatGPT 4.0 and Google Gemini, providing insights into their respective strengths and weaknesses for their potential use in an educational setting. This analysis is intended to contribute a valuable perspective to the ongoing discussion about the optimal use of language learning models in various fields.

## Materials and methods

**Figure 1:**
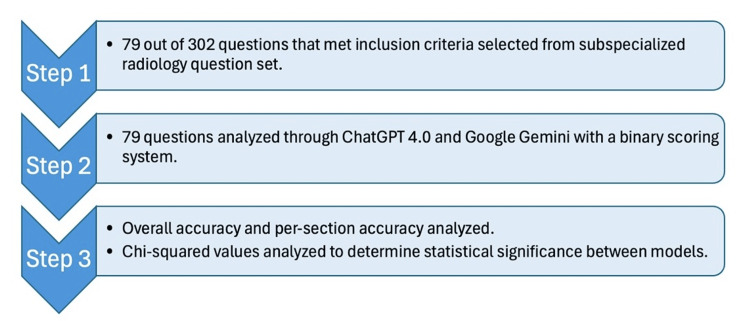
Series of steps within methods followed in this study

This study analyzed the accuracy of ChatGPT 4.0 and Google Gemini versions as of August 2024. Our primary objective was to determine if there was statistical significance in the overall accuracy of each AI model when answering standardized questions from a pediatric radiology question set [12]. To establish our inclusion criteria, we included questions that were text-only and excluded image-based questions. From the textbook's total of 302 questions, we selected the 79 questions that met these criteria. Of these 79 questions, 38 were slightly modified by the authors to remove references to prior answers and adhere to the text-only format. For example, an unedited question from the textbook that read, "Regarding the most likely diagnosis of the patient in Question 8, which of the following is true?" was changed to "Regarding [specific medical condition], which of the following is true?" This alteration removed the reference to a previous question while maintaining the core concept being tested. These 79 questions were drawn from seven subsections, each representing a distinct field such as musculoskeletal system, and chest radiology (Figure 1). Multi-disciplinary questions were excluded to create a better analysis of accuracy by a specific subsection. Accordingly, our secondary objective sought to determine the presence of statistical significance between each AI model’s accuracy within individual subsections. 

**Figure 2:**
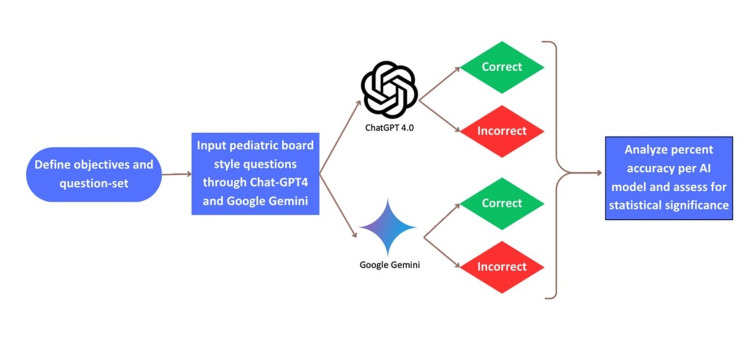
Illustrative diagram outlining sequences within the methodology

During the collection of data, questions were answered using a binary scoring system where correct responses were marked as 1 and incorrect responses as 0. After running the questions as individual inputs, multiple chi-square analyses were utilized to determine the difference between the two models. We analyzed the results to identify statistical significance, qualifying p-values to be significant with a cut-off of 0.05 and calculating relative risk (RR) with 95% CI. The results were further analyzed across subsections to assess accuracy (Figure 2). Since this study did not involve patient data, Institutional Review Board (IRB) approval was not required. This allowed for a streamlined research process, focused solely on evaluating the performance of the AI models under controlled conditions. All the study’s data and analyses were securely recorded in a Microsoft Excel (Microsoft Corporation, Redmond, Washington, USA) spreadsheet and verified for accuracy. The data was verified for accuracy through a comprehensive process, where two researchers independently entered the data into separate Excel spreadsheets for calculations. We then used an automated comparison via Excel to identify any discrepancies between the two entries. Following this, the researchers reviewed all calculations and discrepancies manually. Finally, our senior researchers on the team reviewed the final results to ensure their accuracy and reliability.

## Results

**Figure 3:**
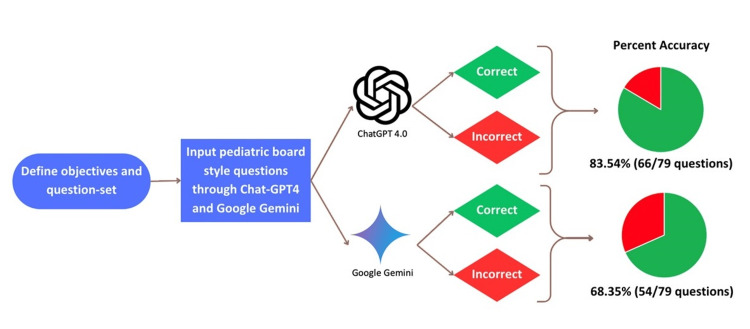
Overall diagnostic accuracy between models

The analysis of ChatGPT 4.0 and Google Gemini reflected a statistically significant difference in overall accuracy. ChatGPT 4.0 answered 83.5% of the 79 questions correctly, while Google Gemini answered 68.35% correctly (Figure 3). This difference in performance was evident in specific questions. For example, when asked "Regarding neuroblastoma stage IV-S, which of the following is TRUE?", ChatGPT 4.0 correctly answered "B. It affects the skin, liver, and bone marrow," while Google Gemini incorrectly responded, "D. There is no metastatic disease." Similar differences were further illustrated within other subsections as well.

The chi-square test yielded a chi-squared value of 4.99 with a p-value of 0.0255, and a RR of 1.221 (95% CI: 1.020 to 1.459), demonstrating that ChatGPT 4.0 was 22.1% more likely to provide correct answers than Google Gemini within the overall sample size (Table 1).

**Table 1:** Table delineating sample sizes per subsection, chi-squared values, p-values, RRs, and 95% CI RR, relative risk

Subsection	Number of questions	Chi-squared value	P-value	RR	95% CI
Overall	79	4.99	0.0255	1.221	1.020-1.459
GI tract	13	0.866	0.352	1.223	0.795-1.879
GU tract	14	2.052	0.152	1.393	0.862-2.252
MSK	15	0	1	1	0.827-1.209
Chest	2	1.334	0.248	2	0.501-7.997
Neuroradiology	5	0	1	1	0.827-1.209
Vascular	15	2.222	0.136	1.57	0.842-2.916
Cardiac	15	0.6	0.439	1.222	0.731-2.040

**Figure 4:**
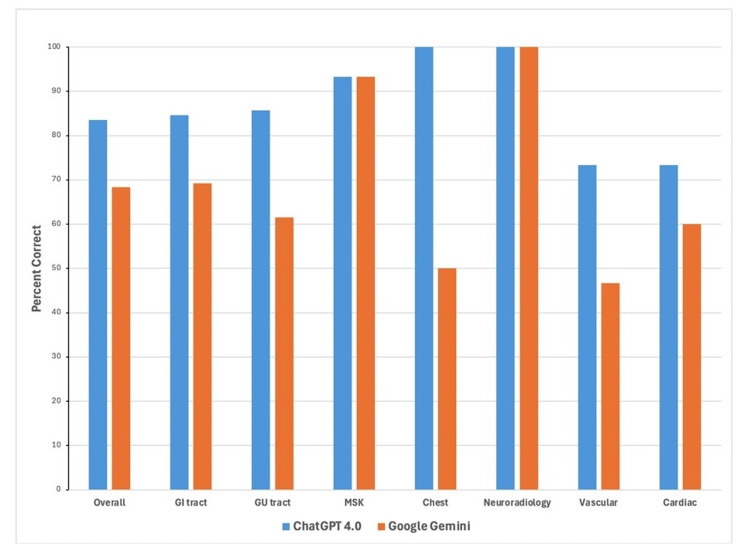
Diagram comparing diagnostic accuracy between ChatGPT 4.0 and Google Gemini overall and within subsections

**Figure 5:**
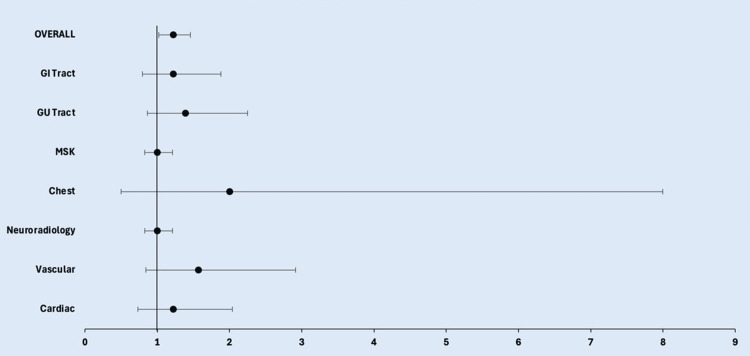
Forest plot (RR with 95% CI) reflecting the lack of statistical significance within subsections between models but reflecting the presence of statistical significance in overall performance RR, relative risk

However, there was no statistically significant difference in accuracy between the two models when the results were analyzed individually by subsection. The closest to statistical significance were the genitourinary tract and vascular radiology, with p-values of 0.152 and 0.136, respectively, and RR values of 1.393 (95% CI: 0.862, 2.252) and 1.570 (95% CI: 0.842, 2.916). In subsequent order of increasing p-values were chest (p-value: 0.248, RR: 2.000 (95% CI: 0.501, 7.997)), gastrointestinal tract (p-value: 0.352, RR: 1.223 (95% CI: 0.795, 1.879)), cardiac radiology (p-value: 0.439, RR: 1.222 (95% CI: 0.731, 2.040)), musculoskeletal system (p-value: 1.000, RR: 1.000 (95% CI: 0.827, 1.209)), and neuroradiology (p-value: 1.000, RR: 1.000, with no meaningful CI due to identical performance) (Figures 4, 5 and Table 1).

## Discussion

Overall accuracy and performance variability

The significant difference in overall accuracy between ChatGPT 4.0 and Google Gemini indicates that ChatGPT 4.0 may be more reliable in answering text-based questions. This reliability could be due to ChatGPT’s specific datasets that help it better answer specific types of questions, compared to Google Gemini’s multimodal training [1, 13, 14]. The RR analysis also supports the data that ChatGPT 4.0 is more likely to answer correctly overall, with an RR of 1.221. However, the lack of statistical significance within subsections, such as in MSK and neuroradiology, may suggest that true significant differences were seen on an aggregate scale that could not be seen perhaps due to the smaller sample sizes within sections. 

Additionally, the exclusion of image-based questions, which are vital in fields such as radiology, may have influenced the results. Including these questions could have provided a more comprehensive comparison, although significant improvements are yet necessary in AI models for an accurate assessment [15, 16]. 

Subgroup performance and analysis

Despite the overall significant difference in accuracy between ChatGPT 4.0 and Google Gemini, our subgroup analysis did not reveal statistically significant differences when examining the results by specific subspecialties. For instance, in the genitourinary tract and vascular radiology, ChatGPT 4.0 demonstrated a trend toward better performance, with RR of 1.393 and 1.570, respectively. However, these differences were not statistically significant, as indicated by p-values of 0.152 and 0.136. Similarly, in chest radiology, ChatGPT 4.0 appeared to outperform Google Gemini, with an RR of 2.000, but this difference also lacked statistical significance (p-value: 0.248).

For other subspecialties, such as the gastrointestinal tract, cardiac radiology, musculoskeletal system, and neuroradiology, the performance of the two models was nearly identical. This was reflected in p-values ranging from 0.352 to 1.000 and RR values that indicated no meaningful differences in accuracy.

These findings suggest that while ChatGPT 4.0 may hold an advantage in overall accuracy, the differences within specific subspecialties were not as pronounced. The lack of statistical significance in these areas could be attributed to smaller sample sizes, which might not have been sufficient to detect subtle differences between the models. This highlights the need for future research to include larger sample sizes within each subspecialty and potentially incorporate image-based questions, which may provide a more comprehensive comparison of these AI models' strengths and weaknesses in specific radiology fields.

Limitations and potential biases

The limitations of this study include the exclusion of image-based questions, utilizing a single subspecialized question set within pediatric radiology, and the low sample size for certain sections, all of which rendered it difficult to determine statistically significant differences in accuracy where they might exist. Moreover, this study did not provide a longitudinal assessment of the AI models' performance, which is relevant given the rapid pace of improvement in these models. The absence of evaluation of the models' performances might evolve over time limiting our understanding of their potential and reliability in clinical settings. Additionally, the lack of comparison to a human radiologist's performance further limits the ability to contextualize these AI models. The implications of these limitations include an incomplete assessment of the models' true performance, particularly given that this study was conducted with a controlled question set rather than real-life patient scenarios involving actual patient data [1, 17, 18].

Future studies should aim to increase the sample size and include an increased number of AI models to provide a more accurate assessment of each model's strengths and weaknesses.

Implications within education

The educational implications of this study are significant in fields such as general or specialized medical education. AI models like ChatGPT and Google Gemini could be utilized in training students with both text and image-based questions [19, 20]. For example, ChatGPT 4.0’s current performance suggests that it could be used to better understand case studies or accurately summarize educational content [18]. The potential of AI in medical education extends beyond radiology, with applications in other specialties where analyzing text is critical [20, 21]. Integrating AI models could further support medical residents or physicians in breaking down complex topics or creating personalized learning experiences [22].

Ethical considerations

The ethical considerations of AI models in educational settings are important, especially in fields such as radiology. Public concern over the use of AI in healthcare has grown, with recent studies indicating that while people realize the potential benefits of AI, they are apprehensive about its accuracy, data privacy, and the potential for bias [16, 19]. In radiology specifically, the concern remains around using AI for decision-making in a field where errors could have serious consequences [19, 22]. This study's findings underscore the importance of methodically evaluating various AI models before their adoption in fields such as healthcare or general education [23].

Furthermore, the integration of AI in radiology education (or within any field) raises questions about developing the critical thinking skills of trainees. There is a need to find a balance between utilizing AI as an educational tool and ensuring that future medical professionals develop and maintain the skills needed for independent clinical judgment.

## Conclusions

The study demonstrates a statistically significant difference in accuracy between ChatGPT 4.0 and Google Gemini when answering standardized radiology-related questions, with ChatGPT 4.0 achieving an accuracy rate of 83.5% compared to Google Gemini's 68.4%, suggesting that ChatGPT 4.0 may be more reliable for certain text-based tasks in medical education. However, the observed variability across different pediatric radiology subspecialties and the exclusion of image-based questions indicate that both AI models have distinct strengths and weaknesses that should be carefully considered. The findings emphasize the potential role of AI in enhancing medical education and diagnostic capabilities, particularly in radiology, while also underscoring the need for responsible integration of these technologies to complement human expertise. Future research should focus on refining these models and developing guidelines for their ethical and effective use in healthcare and educational contexts.
